# Early Detection of Exposure to Toxic Chemicals Using Continuously Recorded Multi-Sensor Physiology

**DOI:** 10.3390/s21113616

**Published:** 2021-05-22

**Authors:** Jan Ubbo van Baardewijk, Sarthak Agarwal, Alex S. Cornelissen, Marloes J. A. Joosen, Jiska Kentrop, Carolina Varon, Anne-Marie Brouwer

**Affiliations:** 1 Department Human Performance, The Netherlands Organisation for Applied Scientific Research (TNO), 3769 DE Soesterberg, The Netherlands; jan_ubbo.vanbaardewijk@tno.nl (J.U.v.B.); rk.sarthak01@tgmail.com (S.A.); 2 Circuits and Systems (CAS) Group, Delft University of Technology, 2628 CD Delft, The Netherlands; j.c.varon@tudelft.nl; 3 Department CBRN Protection, The Netherlands Organisation for Applied Scientific Research (TNO), 2288 GJ Rijswijk, The Netherlands; marloes.joosen@tno.nl (M.J.A.J.); jiska.kentrop@tno.nl (J.K.)

**Keywords:** electrocardiography, electroencephalography, respiration, machine learning, chemical exposure, toxidrome detection, differential diagnosis, opioid, nerve agent

## Abstract

Early detection of exposure to a toxic chemical, e.g., in a military context, can be life-saving. We propose to use machine learning techniques and multiple continuously measured physiological signals to detect exposure, and to identify the chemical agent. Such detection and identification could be used to alert individuals to take appropriate medical counter measures in time. As a first step, we evaluated whether exposure to an opioid (fentanyl) or a nerve agent (VX) could be detected in freely moving guinea pigs using features from respiration, electrocardiography (ECG) and electroencephalography (EEG), where machine learning models were trained and tested on different sets (across subject classification). Results showed this to be possible with close to perfect accuracy, where respiratory features were most relevant. Exposure detection accuracy rose steeply to over 95% correct during the first five minutes after exposure. Additional models were trained to correctly classify an exposed state as being induced either by fentanyl or VX. This was possible with an accuracy of almost 95%, where EEG features proved to be most relevant. Exposure detection models that were trained on subsets of animals generalized to subsets of animals that were exposed to other dosages of different chemicals. While future work is required to validate the principle in other species and to assess the robustness of the approach under different, realistic circumstances, our results indicate that utilizing different continuously measured physiological signals for early detection and identification of toxic agents is promising.

## 1. Introduction

Exposure to chemical agents, e.g., in military or industrial contexts, can lead to life-threatening conditions. Quick detection of exposure to such a chemical, followed by differential diagnosis, is paramount in enabling timely (self-administered) treatment; dependent on the type of chemical and route of exposure, intoxication can incapacitate within minutes. Currently, diagnosis of an exposure is based on appearance of clinical symptoms, the latter being dependent on the presence of medically trained professionals, followed by verification of exposure by specialized equipment. However, in many situations, differential diagnosis to initiate appropriate medical countermeasures may be too late.

In order to accelerate the detection of exposure, as well as the identification of the nature of the chemical agent at hand, we may exploit recognition of physiological changes. Dependent on the chemical class and the type of exposure (e.g., through inhalation or through the skin), unique profiles of change in different physiological variables are expected. Such profiles may be detectable before overt symptoms appear. Recent advances in wearable sensors have improved user comfort, biological compatibility, signal quality, and enabled monitoring multiple physiological parameters simultaneously [1,2,3,4,5]. Wearable sensors are developed and applied in the context of monitoring patients with certain suspected underlying conditions. They can be invaluable tools for diagnosing pathological conditions, such as arrhythmias and epilepsy, which may only surface occasionally and thus might easily be missed during a doctor’s visit [6]. Continuous physiological measurements can be useful for other purposes as well, such as monitoring general health and mental state [7,8,9], which are especially relevant in high risk (military) professions. This means that these wearable sensors may be in place already and that minor adaptations could broaden the applicability towards diagnosis of exposure to chemical agents.

Concerning the use of wearable sensors to detect specific chemical agents, such sensors have been successfully used to continuously monitor alcohol intake by analyzing alcohol levels in sweat [10,11]. Additionally, in cases where it is not possible to directly measure the chemical of interest itself, wearable sensors have been shown to be potentially useful for the detection of exposure to chemicals, based on the chemicals’ physiological and behavioral effects [12,13,14]. These studies investigated the feasibility of using physiological and locomotion information from wearable sensors to detect opioid intake. Users wore a wristband that monitored electrodermal activity, skin temperature and acceleration. A machine learning model was used successfully to automatically detect opioid intake based on features derived from these physiological variables [13]. Relevant features were skin temperature and locomotion.

In the current study, and as a first step towards detecting intoxication following chemical exposure in humans using wearable sensors, we assessed detection and identification of acute exposure to two distinct potent chemicals in guinea pigs using continuously recorded electrocardiogram (ECG), electroencephalogram (EEG) and respiration in combination with machine learning techniques. The chemicals used were an opioid, fentanyl, and a nerve agent, VX, both potent chemicals that could be weaponized and used as warfare agents by state actors or terrorist organizations [15,16]. Fentanyl and VX were used as model compounds to study exposure scenarios involving inhalational exposure to a potent opioid, or skin exposure to a nerve agent, respectively. Including both these chemical classes is interesting because differential diagnosis may be hampered as a result of overlap in symptoms [17]. Fentanyl is a highly potent synthetic opioid, with clinical applications as an analgesic and sedative [18]. The major physiological effects associated with fentanyl use are opioid-induced respiratory depression (OIRD), caused by central and peripheral inhibition of the respiratory drive [19,20], and bradycardia [21]. VX is a low volatile organophosphate nerve agent. Exposure leads to cholinergic crisis, associated with various muscarinic (salivation, bronchorrhea, bradycardia, emesis) and nicotinic (paralysis, sweating, fasciculations) symptoms. These symptoms, in combination with secondary respiratory depression, may become fatal in severe poisoning cases [22,23]. Skin contamination presents a major risk for VX, due to its low volatility and persistence. VX forms a skin depot, through which it readily penetrates into the circulation and requires rigorous and continuous treatment [24,25,26]. The routes of exposure the current study included the intravenous (i.v.), subcutaneous (s.c.), and percutaneous (p.c.) routes, representing both rapid (i.v., s.c.) and gradual (p.c.) intoxication scenarios.

In this study, a machine learning approach (LSTM neural network) was employed in which a model was first trained and tested for *detecting* exposure to either of the chemicals. A second model was used to *differentiate* between fentanyl and VX. Additionally, the *timeline* of detection and differentiation after exposure was evaluated. To determine the *relative importance of ECG, EEG and respiration*, models were trained and tested using different sets of features derived from (combinations of) the three sensors. When studying and modelling detection of toxic agents, *generalization* of concepts is important since toxic agents, which could be used in chemical warfare, cannot be experimentally studied in humans. Here, it was examined whether models trained on a certain chemical exposure in animals, could be used to detect exposure involving different conditions regarding the type of chemical, dosage and route of administration.

## 2. Materials and Methods

### 2.1. Data

Four existing physiological datasets of guinea pigs, exposed to VX, fentanyl, or placebo, were used. Animal procedures were described previously [27]. VX was obtained from the TNO stocks. Purity was checked upon issue and was >98%. Fentanyl citrate (European Pharmacopoea grade) was purchased from Spruyt-Hillen (IJsselstein, The Netherlands). Purity was >99%. The VX doses used were 1–2 mg/kg (percutaneous), corresponding to approximate 1.5–3 times the 24 h LD50 values in guinea pigs [28]. The fentanyl doses ranged from 0 (placebo) to 32 mg/kg (intravenous or subcutaneous) and were selected to elicit varying degrees of respiratory depression, up to lethality. Fentanyl was dissolved in phosphate-buffered saline (PBS) to the required concentration before administration. VX was either dissolved in 2-propanol (IPA) to the required concentration or directly applied as neat agent. All experiments were carried out according to the EU Legislation for testing on experimental animals (EU Directive 2010/63/EU) at the TNO CBRN Protection Department, Rijswijk, The Netherlands.

#### 2.1.1. Exposure and Recording

Table 1 gives an overview of the chemical exposure in the four sets of animals that were examined in this study. Animals were exposed to VX percutaneously, or to fentanyl intravenously or subcutaneously, with varying dosages. Control (placebo) animals were included in the Fentanyl datasets. These animals received vehicle solution treatment consisting of PBS.

For continuous physiological measurements, animals were surgically equipped with ECG and EEG leads. For ECG analysis, two leads were sutured in the superficial muscles under the skin right below the right collar bone and between the second and third rib (configuration II). For cortical EEG analysis, two leads were secured at the dura mater (A8.0 and P1.2 mm relative to bregma and 1 mm from the sagittal suture). ECG and EEG data were transmitted wirelessly to a hardware system (Data Sciences International) using F40-EET or HD-S02 transmitters at sampling rates of 240 Hz and 375 Hz, respectively. Unrestrained respiratory plethysmography (URP) data were obtained using whole-body plethysmography cages (Data Sciences International), connected to a Universal XE signal conditioner. All physiological data were upsampled at 1000 Hz and processed using Ponemah (v5.41) and NeuroScore (v3.3.1) for ECG/URP and EEG data, respectively. Artefacts related to disturbances in ambient surroundings were manually excluded. This concerned <2% of the data. For each animal, at least 30 min of baseline data were acquired before exposure.

#### 2.1.2. Extracted Parameters

All ECG/URP data were divided into epochs of 6 s duration (VX sets), 10 s or 60 s duration (Fentanyl sets). For each epoch, the average values were calculated for different parameters. For ECG, these were heart rate (HR), QT-interval and ST-interval. For URP, these parameters were tidal volume (TV), peak inspiratory flow (PIF) and peak expiratory flow (PEF).

EEG spectral analysis was performed via Fast Fourier Transformation (FFT) over a total bandwidth of 0.5–100 Hz. Epoch duration was set at 10 s intervals. Total power was determined, and power spectra were divided into Delta (0.5–4 Hz), Theta (4–8 Hz), Alpha (8–12 Hz), Sigma (12–16 Hz), Beta (16–24 Hz), and Gamma (24–100 Hz) bands and calculated relative to the total bandwidth.

### 2.2. Pre-Processing of Data for Modelling

MATLAB R2020a was used to pre-process the data as described above, so that it could be used for modelling. Data were divided into non-overlapping one-minute samples, averaging the variables as assessed over 6 or 10 s into one value. For each animal, we selected data 30 min before exposure until 45 min after exposure. We removed 4 min of data around the moment of exposure to prevent any handling effects potentially influencing our dataset. This resulted in a dataset of 71 samples per animal.

All data were obtained in freely moving animals. Signal artifacts, related to movement, were excluded. These missing data points were imputed using the nearest neighbor imputation algorithm [29]. When more than 40% of the data of a particular feature was missing in one animal, that feature was removed entirely and replaced by the average values of that particular feature over all animals. This happened in 7 animals for respiration, in 9 animals for ECG and in 1 animal for EEG, out of a total of 135 animals. Average percentages of imputed data across the four datasets were 6.8, 9.8 and 5.0 for respiration, ECG and EEG, respectively.

Standardization was performed using the mean and standard variance of the first 30 min before (possible) exposure. Thus, output features were centered around the mean of these 30 min with variance 1 ensuring similar feature characteristics across different animals and features.

For all four datasets, samples from before exposure were labelled ‘healthy’. All the samples of animals that received a placebo exposure were labelled ‘healthy’ as well. Samples from animals after exposure to a chemical were labelled with their respective chemical (fentanyl or VX).

### 2.3. Modelling

The type of classification model used was a Long short-term memory (LSTM) model [30]. LSTM is optimized to detect long term dependencies in the data and considers past (variations of) values to predict. For implementation, Python 3.9.0 was used with Tensorflow 2.4 and Keras 2.4.3.

**Figure 1:**
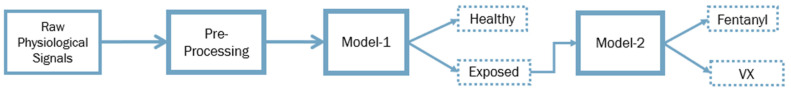
Overview of the study. After pre-processing, Model 1 classifies the samples into either ‘healthy’ or ‘exposed’. Model 2 then classifies ‘exposed’ samples into either ‘fentanyl’ or ‘VX’.

Different models were trained, with different purposes (Figure 1). ‘Model 1’ was trained to distinguish healthy samples (from placebo animals, and from exposed animals before exposure) from exposed (either VX or fentanyl) samples. ‘Model 2’ was trained to differentiate the samples labelled as ‘exposed’ into VX or fentanyl. Both models had the same setup of 5 LSTM cells. The output of the final LSTM cell was passed to a dense layer (input and output shape both equal to 2) with a sigmoid activation function. Hyperbolic tangent and hard sigmoid functions were used for activation and recurrent activation, respectively, for each of the LSTM cells. Dropout regularization [31] was used to avoid overfitting and training was done by the Adaptive moment estimation (ADAM) method [32].

#### 2.3.1. Cross-Validation Approach

The general cross-validation approach that was applied worked as follows. The data samples were split into two sets of animals: a training set and a test set. The test set was stratified so that a proportional number of animals from each of the four datasets was present in the test set. Presence of placebo animals from the fentanyl datasets in both the training and the test set was verified. The training set contained about 80% of the animals, the test set the remaining 20%. Optimization of the parameters was done using cross-validation on the training set, in a repeated random sub-sampling fashion (Monte Carlo). Multiple random splits were made of about 12.5% of the training set as a validation set. This was again done using a stratified variant where each set contained a proportional number of animals from each of the four sets. For each split, the validation data was used to tune the hyperparameters and to decide on the final model parameters. The final model was trained on the whole training set and applied on the independent test set to obtain the final performance score. Table 2 gives an overview of the number of samples and animals involved in the different sets.

#### 2.3.2. Exposure Detection Delay

Inevitably, some time passes between the exposure to the chemical and the chemical’s specific effect on physiology that can be detected by the models. To examine how fast Model 1 detected exposure, and how fast Model 2 recognized the correct chemical, classification accuracy is viewed over the successive one-minute samples, averaged across animals.

#### 2.3.3. Feature Set Performance

We repeated the training and testing procedures of both models with only one of the three different feature sets that correspond to the three sensors as input (ECG features: HR, QT-interval and ST-interval; EEG features: Delta, Theta, Alpha, Sigma, Beta, Gamma, and total power; respiratory features: TV, PIF and PEF), and the different possible combination of sensors. We performed this procedure twice: once during cross-validation on the validation set with the purpose of selecting which feature sets we want to use for that particular model as a default, and once at the end on the test set with the purpose of determining the relative feature importance for that model. Table 3 shows the results on the validation set. Based on these results, only ECG and respiration features were chosen to be used in the default Model 1 (i.e., a total of six features), and only EEG features for default Model 2 (i.e., seven features).

#### 2.3.4. Generalization

To assess to what extent Model 1 generalized from one dataset to other datasets, with differences in chemicals and dosages, separate models were trained using one of the four datasets. Each of these models was then tested on each of the other datasets as test sets.

#### 2.3.5. Animal Classification

The results of Model 2 were also used to move from the sample level to the level of an animal: for each animal, based on the sample results, we predicted whether this animal was exposed to either VX or fentanyl. For each of the animals, the percentage of samples in that window that were predicted as either ‘VX’ or ‘fentanyl’ by Model 2 was assessed and, based on that percentage, the animal was labeled as either exposed or healthy. Note that a similar analysis of translating results from the sample level to the animal level was not done for Model 1, because of the low proportion of placebo animals (see Table 1).

## 3. Results

The resultant accuracies were 97.84% correct detection of exposure with Model 1 and 94.14% correct differentiation between VX and fentanyl with Model 2, both achieved on the independent test set.

**Figure 2:**
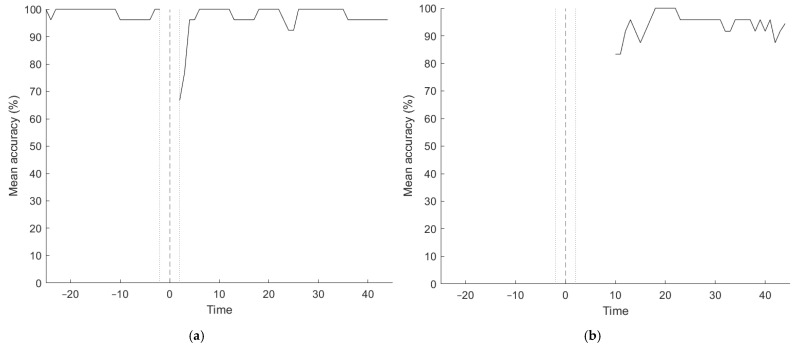
Classification accuracy (%) for (**a**) Model 1 (exposure detection) and (**b**) Model 2 (chemical differentiation) as a function of elapsed time (minutes). The dashed line marks the start of exposure, the dotted lines mark the four minutes around exposure where were was removed due to potential handling effects.

Figure 2 shows the accuracy of the models as a function of time. For Model 1, exposure detection accuracy is high before exposure, indicating that the model correctly classifies healthy samples. Accuracy drops immediately after exposure, indicating that exposure is not detected at once. About 5 min after exposure, samples are classified correctly as ‘exposed’. Differentiation between chemicals for those samples that were classified as exposed (starting at the tenth minute after exposure) is over 90% correct for almost all time samples.

Table 4 shows the results of the feature importance analysis on the test sets. As found for the validation set, classification accuracies when features of single sensors are used show that respiratory features are most important for Model 1, and EEG features are most important for Model 2. Additionally, consistent with the validation results, adding ECG features to respiratory features in Model 1 (resulting in the default model) improved performance. Again, for Model 2, including other features besides EEG did not improve performance, but rather decreased it.

Table 5 shows the results for the generalization of Model 1 across datasets. Models were trained with data from one of the four datasets (rows in Table 5), and tested on each of the other three datasets (columns in Table 5), to explore how well the trained models generalize to other (types of) datasets. Models trained on one fentanyl dataset perform very well when tested on the other fentanyl dataset and models trained on one VX dataset perform well when tested on the other VX dataset (accuracies between 92% and 98% correct). However, models trained on VX also perform well on fentanyl datasets (accuracies 84%–91%), and models trained on fentanyl performed well on the VX2 dataset (92% and 95% correct). Detection of exposure in the VX 1 dataset was relatively difficult when models were trained on the fentanyl datasets.

For Model 2, the predictions on animal level resulted in an accuracy of 95.83% correct (23 correctly classified from 24 animals). This means that for each exposed animal most of the samples were predicted correctly as either VX or fentanyl.

## 4. Discussion

In the current study, machine learning models (LSTM neural networks) were trained on physiological data obtained from a heterogeneous sample of freely moving, chemical-exposed guinea pigs, with the aim to detect exposure to chemicals in new, unseen animals, and in case of exposure, to identify which of two chemicals (fentanyl or VX) the animal was exposed to. Detection of exposure was possible with an accuracy of over 95% already 5 min following exposure. Correct differentiation between fentanyl and VX was possible with an accuracy of almost 95%. Physiological features from the three different types of sensors were differentially important depending on whether the purpose was the detection of exposure (where respiration features proved to be most important) or chemical differentiation (EEG features were most important). It is not feasible to obtain data for training models to detect exposure to dangerous chemicals from human experiments due to ethical reasons. It is therefore promising that the models generalized across chemicals and dosages because training data may then be obtained using other chemicals (e.g., in the case of nerve agent chemicals that have a reversible effect, such as a carbamate) or lower, safe dosages.

We believe that our study illustrates the innovative and potentially valuable concept of using physiology for early and automatic chemical exposure detection in a single individual, such as a military professional. However, our study is limited when considering this ultimate goal, and a number of questions are open for future research as outlined in the paragraphs below.

Physiological measurements were performed using equipment that is not likely to be used in humans. As discussed in the introduction, the vision is to use physiological variables recorded using wearable sensors. While wearable sensors for measuring heart rate, respiration and EEG exist, signal quality is expected to be lower in such devices compared to implanted sensors and the whole-body plethysmographs used. On the positive side, additional potentially informative sensors can be included, most notably movement sensors.

As discussed, the successful generalization across datasets indicates that detection of exposure can be robust across conditions involving different dosages and types of chemicals. These results suggest that, also in humans, generalization from detecting chemical exposure following lower doses, leading to less physiological changes, may generalize to the early detection of exposure to incapacitating or lethal doses of chemicals. This is promising for the eventual translation to the human application. Results for highly toxic chemicals can be derived from animal experiments, preferably from data sets that have already been collected for other purposes to minimize animal use. Furthermore, future work should examine features that are unaffected by variation in factors such as species or physical stress. It might be that the interaction between physiological measures in healthy conditions and exposed conditions is relatively constant.

Future studies should also consider the longer timelines and the imbalance of the amount of ‘healthy’ and ‘exposed’ data. The current datasets contained very few placebo animals and a roughly equal amount of healthy and exposed data. Data were baselined using healthy data and, by definition, exposed data always occurred after healthy data. Even though all datasets showed stable baseline behavior, exposure detection may have been influenced by time-varying factors influencing physiology. Given the accurate performance on placebo animals, this is not expected to be a major problem but, for future studies, more placebo animals, or more healthy data, would be desirable.

## 5. Conclusions

We conclude that the present study yielded a promising approach to explore development of machine learning models using physiological data from wearable sensors that could provide an early trigger for countermeasures in case of exposure to a highly toxic chemical. Quick, automatic diagnosis will allow a crucial increase in the time window in which effective interventions may be administered. These results showed that quick detection and differential diagnosis may be feasible, allowing for agent specific treatment which would further increase the lifesaving potential of medical countermeasures.

## Figures and Tables

**Table 1:** Overview of chemical exposure in the four datasets. While most animals in the dataset ‘Fentanyl subcutaneous’ were exposed to Fentanyl subcutanenously, 6 animals were exposed intraveneously.

Exposure	Total nr of Animals	Nr of Placebo	Dosage	Vehicle/Volume
Fentanyl intravenous	26	6	0–16 mg/kg	PBS/1 mL/kg
Fentanyl subcutaneous	45	6	0–32 mg/kg	PBS/1 mL/kg
VX percutaneous (1)	32	0	1 mg/kg	IPA/16 µL/kg
VX percutaneous (2)	32	0	2 mg/kg	Neat/1 µL

**Table 2:** Number of samples (number of animals) in the various sets for Model 1 and Model 2.

Model	Training Set	Validation Set	Testing Set	Total Set
1	6598 (93)	1136 (16)	1844 (26)	9578 (135)
2	3699 (85)	565 (13)	1042 (24)	5306 (122)

**Table 3:** Model 1 and Model 2 classification accuracy for different combinations of features, defined by sensor. The bold and underlined percentages mark the chosen default feature sets.

Feature Set Included	Model 1 Validation Set: Mean Accuracy (%)	Model 2 Validation Set: Mean Accuracy (%)
EEG	73.67	**98.12**
ECG	81.48	79.24
Respiration	93.13	84.00
EEG + ECG	87.85	97.29
EEG + respiration	92.67	95.07
ECG + respiration	**95.21**	89.98
EEG + ECG + respiration	92.03	91.32

**Table 4:** Model 1 and Model 2 classification accuracy for combinations of features, defined by sensor. Classification accuracy of the default models is bold and underlined.

Feature Set Included	Model 1 Test Accuracy (%)	Model 2 Test Accuracy (%)
EEG	75.21	**94.14**
ECG	81.97	84.81
Respiration	94.22	89.58
EEG + ECG	84.85	92.19
EEG + respiration	94.23	93.60
ECG + respiration	**97.84**	88.33
EEG + ECG + respiration	96.13	94.03

**Table 5:** Generalization of Model 1 across datasets.

Accuracy (%)		Tested on…			
		Fentanyl i.v.	Fentanyl s.c.	VX 1	VX 2
Trained on…	Fentanyl i.v.	-	97	68	95
	Fentanyl s.c.	98	-	60	92
	VX 1	84	89	-	95
	VX 2	89	91	92	-

## Data Availability

The data presented in this study are available on request from the corresponding author. The data are not publicly available due to obligations imposed by the sponsor of the original studies.
